# Polymeric Protective Films as Anticorrosive Coatings—Environmental Evaluation

**DOI:** 10.3390/polym16152192

**Published:** 2024-08-01

**Authors:** Alina Ruxandra Caramitu, Romeo Cristian Ciobanu, Magdalena Valentina Lungu, Eduard-Marius Lungulescu, Cristina Mihaela Scheiner, Mihaela Aradoaei, Adriana Mariana Bors, Traian Rus

**Affiliations:** 1 National Institute for Research and Development in Electrical Engineering ICPE—CA Bucharest, 030168 Bucharest, Romania; alina.caramitu@icpe-ca.ro (A.R.C.); magdalena.lungu@icpe-ca.ro (M.V.L.); marius.lungulescu@icpe-ca.ro (E.-M.L.); 2 Department of Electrical Measurements and Materials, Gheorghe Asachi Technical University, 700050 Iasi, Romania; cristina-mihaela.schreiner@academic.tuiasi.ro (C.M.S.); mihaela.aradoaei@academic.tuiasi.ro (M.A.); 3 National Research and Development Institute for Optoelectronics INOE 2000—IHP Bucharest, 077125 Măgurele, Romania; bors.ihp@fluidas.ro; 4 Department of Inorganic Chemistry, Physical Chemistry and Electrochemistry, National University of Science and Technology—Politehnica Bucharest, Splaiul Independentei No. 313, 060042 Bucharest, Romania; traianrus@hotmail.com

**Keywords:** polymeric coatings, corrosion protection, defraud factors

## Abstract

The behavior of two polymeric protective paint coatings (epoxy and polyurethane) applied over an epoxy primer coating on steel plates was investigated in this study, focusing on their role in providing anticorrosive protection against various climatic stress factors. Among the numerous climatic factors that can affect the lifetime of anticorrosive coatings, the following were selected for this work: dry heat, UV radiation, humidity, and extreme conditions such as salt fog, marine atmosphere, and alpine atmosphere. The objective was to determine the remaining lifetime of these protective coatings before replacement is needed to prevent damage to the equipment they protect. The behavior of these polymeric materials under the mentioned factors was analyzed based on the variation in the tangent of the dielectric loss angle (tg δ) with frequency. From the interpretation of the experimental results, it was found that the polyurethane paint coating (P2) exhibits superior resistance to climatic degradation compared to the epoxy paint coating (P1). Furthermore, a comparison of tg δ values for the P1 and P2 coatings revealed that the initial (unaged) P2 coating performs better as an insulator (dielectric) than the P1 coating. Comprehensive information is provided to the users of polymeric anticorrosive protection materials, highlighting the extent to which climatic factors can affect the performance of the equipment they protect and determining the appropriate timing for replacing the coatings.

## 1. Introduction

Significant economic losses are caused by the corrosion of materials, mainly due to the repair and replacement of damaged materials, the use of more corrosion-resistant materials, the application of expensive coating materials, reduced efficiency, over-design, and pollution. Serious problems are presented by corrosion and wear for various materials used in different industries [1,2,3]. Therefore, a cost-effective, sustainable, and ecological approach to reducing metal corrosion is urgently needed, especially in the steel industry [1,2,3]. Moreover, the carbon dioxide (CO_2_) emissions from producing the steel needed to replace corroded steel are projected to account for 4.1–9.1% of the total emissions by 2030, considering the European Union and U.S. greenhouse gas (GHG) reduction targets [4].

Currently, significant concern exists about finding the most efficient method to mitigate the economic impact of corrosion [5,6,7]. Implementing corrosion prevention and control (CPC) planning is recommended as a key management tool. This approach effectively addresses and reduces the impact of corrosion at every stage throughout a product’s or facility’s life. Technical standards and guidelines [8,9] suggest the following steps in maintenance planning: (i) a condition assessment survey should be planned and conducted; (ii) potential maintenance painting options should be listed; (iii) the economics of the available options should be evaluated; (iv) appropriate maintenance painting options should be selected and implementation procedures established; (v) the selected maintenance painting options should be implemented; (vi) follow-up activities should be planned and implemented.

The physical–chemical, dielectric, and mechanical characteristics of these materials, including melting temperature, modulus of elasticity, density, chemical reactivity, electrical and thermal conductivity, hardness, mechanical resistance to breaking, elongation, and tenacity, are determined by their chemical structure, electronic distribution, spatial configuration, and structural defects [10,11,12].

The durability of polymeric protective coatings is known to be limited, and their remaining functional lifetime depends on the physico-chemical properties of the polymer materials, the type and intensity of degradation factors, and the interactions between them [10,11,12]. To identify an effective anticorrosive polymeric protective film, determining the moment of onset of degradation is very important. This determination allows the estimation of the durability of the coating in a given environment, the monitoring of the degradation phenomenon once it has been initiated, and the prediction of the time when any correction is required, as well as the application of preventive measures [11,12].

Anticorrosive protection is only ensured if very good adhesion properties are obtained between the metallic substrate and the polymeric coatings. An adequate degree of adhesion and, consequently, increased durability of the anticorrosive protection system can be achieved through proper surface preparation of the metallic substrate (cleaning, polishing, etc.) and the appropriate choice of protective coatings [12,13,14].

Metal structures are typically protected with modern anticorrosive coatings consisting of multilayers of different materials applied to the metal surface requiring protection. A primer is usually applied as the initial layer, improving adhesion between the coatings and significantly affecting the electrochemical and diffusion processes at the metal–coating interface [15,16,17,18]. The functional properties of the primer are determined by its chemical composition and depend on the nature and surface quality of the metal substrate [15].

A continuous concern exists at the international level for the manufacture of new durable coatings, necessitating the development of rapid methods for evaluating the corrosion resistance of metal/polymeric coating systems to choose the optimal variant of the polymeric film for anticorrosive protection [12,13,14]. Typical accelerated aging tests to determine the protective capacities of anticorrosive protective polymeric films are based on subjecting the materials to different stress categories (temperature, humidity, ultraviolet (UV) radiation, and salt spray (fog)) at higher levels than those found in the natural environment to induce an accelerated deterioration of the system [14,15,16,17,18,19,20,21,22,23,24,25,26,27,28,29,30,31,32,33,34,35,36,37,38,39,40,41,42].

Advances in anticorrosive paint coatings for steel used in marine environments with a high content of chloride ions have been presented in numerous studies [43,44,45,46,47,48]. For instance, a multifactor coupling accelerated test for anticorrosive coatings in marine environments was employed by Gao et al. [45] using a UV/salt spray coupling test with conditions correlated to hot and humid oceanic climate data. On the other hand, the performance of anticorrosive paint coatings for steel used in alpine environments is rarely discussed in the literature [49].

Polymer-coated steel substrates are usually visually inspected, both before and after exposure to various accelerated conditions, using the naked eye, optical microscopy (OM), and scanning electron microscopy (SEM). This method allows for the easy observation of blistering, rusting, delamination, and other defects in the polymeric coatings. High-resolution images of the coating surface in both longitudinal and cross-sectional views are provided by SEM analysis, revealing defects, cracks, and other morphological features [14,38]. Elemental analysis to detect the presence of corrosion products and the extent of rusting is enabled by energy-dispersive X-ray (EDX) spectroscopy [14]. The chemical changes in the polymeric coatings after exposure to corrosive environments are studied using Fourier Transform Infrared (FTIR) spectroscopy. Specific functional groups affected by corrosion can be identified by analyzing FTIR spectra [38].

The corrosion resistance of coated metals and the onset of degradation in polymeric coatings have long been evaluated using electrochemical impedance spectroscopy (EIS). Information on the corrosion resistance and capacitance of coatings, as well as the integrity of the polymeric coating-metal interface, is provided by EIS spectra. High coating resistance is indicated by good barrier properties, while changes in capacitance suggest coating degradation. Early signs of coating degradation can be detected by EIS before macroscopic defects appear. The effects of extreme temperatures and temperature changes on polymeric coatings have been the focus of many EIS studies [19,20,21].

The degradation of organic coatings due to mechanical factors, such as tensile stress and abrasion, has been identified using EIS [22,23]. Monitoring polymeric coating degradation under accelerated aging conditions like UV radiation, mechanical deformation, and thermal cycling has also been achieved with EIS [16,24,25,26,27,28,29,30,31,32]. The susceptibility of paint systems to degradation in 3 wt.% NaCl solution at ambient and elevated temperatures was classified by Oliveira et al. using EIS [32,34].

Potentiodynamic polarization (PDP) is used to determine the corrosion potential (E_corr_), corrosion current density (I_corr_), and corrosion rate (R_corr_) to understand the electrochemical behavior of polymeric-coated metals. Better corrosion resistance of the tested materials is indicated by lower I_corr_ and R_corr_ values. In EIS and PDP tests, the coated metal plates are typically mounted in resin with 1 cm^2^ exposure to an electrolyte solution (e.g., 3.5 wt.% NaCl solution) [14]. Other qualitative and quantitative methods for evaluating corrosion behavior in polymeric coatings are summarized in [14].

The lifetime of polymer-based protective coatings has been predicted using EIS in numerous studies. In some cases, the lifetime has been correlated with EIS measurements performed under different exposure conditions. In other cases, correlations have been made between EIS measurements and accelerated aging conditions or mechanical properties. For instance, an attempt to predict the lifetime of intact and defective polymeric coatings using EIS was made by Van der Weijde et al. [35]. It was concluded that predicting the lifetime of polymeric protective structures is a complex phenomenon, and EIS can only evaluate the effect in cases of small variations in the factors influencing the lifetime of polymeric coatings.

The ISO 12944-5 standard [36] is the only standard providing direct correlations between accelerated exposure tests and the lifetime of protective polymeric coatings. Categories of different corrosive environments are defined by this standard based on EIS test results. However, EIS is limited to laboratory evaluations and cannot provide information about the lifetime of coating systems under real conditions or in operation. This method only assesses the anticorrosive resistance of protective polymeric coatings.

Studies and experiments in this field have been conducted by many research groups, including specialists from ICPE-CA, and their findings have been presented at specialized events [37,38,39,40,41]. Unlike destructive methods such as EIS and PDP, which are applied to small-sized samples, the dielectric spectroscopy (DS) method is non-destructive and provides comprehensive information regarding the dielectric characteristics of larger polymeric protection systems. This information is essential for evaluating the lifetime of these systems and estimating their performance in operation, allowing for accurate predictions of the moment when the anti-corrosion protection needs to be renewed.

The selection of polymeric paints should be based on comprehensive and reliable information about available products in the market. While manufacturers provide fundamental details on the characteristics and applications of their paints, there is a significant need for independent research to validate manufacturer claims, compare various products, uncover new insights, and assess specific application contexts. 

An objective comparison of different polymer paints as anticorrosive coatings was aimed for in our study, providing unbiased data on their performance. Practical methods using dielectric spectroscopy were developed to select suitable anticorrosive protection polymeric materials after exposure to specific environmental conditions. Through this research, new insights into corrosion resistance were provided, revealing information that manufacturers may not have explored or disclosed. Additionally, specific contexts not covered by general manufacturer guidelines were addressed.

## 2. Materials and Methods

### 2.1. Materials

For our research, commercial paints based on epoxy resin (SigmaCover 456, PPG, Pittsburgh, PA, USA) and polyurethane resin (SigmaDur 550H, PPG) were selected due to their recognized durability, chemical resistance, and suitability for industrial steel substrates. An epoxy anticorrosive primer (SigmaCover 280, PPG) was also used to enhance adhesion and corrosion resistance. The coatings were applied as follows: P1 for epoxy paint/epoxy primer/steel plates, P2 for polyurethane paint/epoxy primer/steel plates, and P3 for epoxy primer/steel plates. Thinners were used as recommended by the manufacturer: PPG Thinner 91-92 for SigmaCover 280 and SigmaCover 456, and PPG Thinner 21-06 for SigmaDur 550H, mixed in volume ratios of base to hardener as 80:20, 82:18, and 87:13, respectively. The choice of paints aligns with industry standards and ensures a reliable comparison under controlled experimental conditions, allowing for an effective evaluation of the performance and practical applications of these coatings.

### 2.2. Equipment and Methods

**Figure 1:**
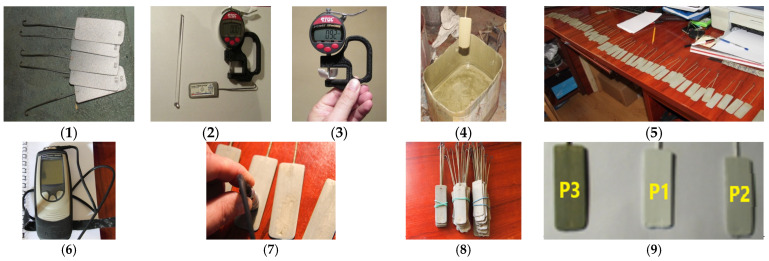
Stages of obtaining samples: (**1**) sandblasting the steel plates; (**2**) determining the surface roughness using the Testex tape method; (**3**) determining the surface roughness to select the proper correction coefficient for measuring dry film thickness (DFT) according to ISO 19840:2012 [42] recommendation; (**4**) painting by immersion in SigmaCover 280 primer; (**5**) [reparing the steel test plates; (**6**) using the DeFelsko Positector 6000 DFT Gauge; (**7**) measuring DFT on both sides of each sample; (**8**) dividing the primer coated plates into three sets with thicknesses as close as possible; (**9**) the three sets of final coated steel plates: Set 1 (P3 coating): SigmaCover 280 green coating/steel plates; Set 2 (P1 coating): SigmaCover 456 white top coating/SigmaCover 280 mid coating/steel plates; and Set 3 (P2 coating): SigmaDur 550H light gray top coating/SigmaCover 280 mid coating/steel plates.

The first step in obtaining polymeric paint coatings involved the successive deposition of a protective layer of epoxy primer paint (plates coded P3) onto steel substrates with length × width × thickness of 68 mm × 26 mm × 1 mm. Following this, a second layer of either epoxy paint (plates coded P1) or polyurethane paint (plates coded P2) was applied over the epoxy primer-coated plates (P3) to achieve a dry film thickness (DFT) in the range of 280–330 μm. The stages for preparing the test samples are shown in Figure 1. 

The paint materials were applied onto steel substrates under controlled environmental conditions: a temperature of 23 ± 2 °C and a relative humidity of 40 ± 3%. Each sample was allowed a minimum curing time of 10 days.

The correction factor, according to ISO 19840 [42], is 10 µm for a surface roughness value for the ten-point height of irregularities Rz = (92–50) µm = 42 µm, where 50 µm is the thickness of the Testex tape, which is deducted from the total value. After the DFT of the P3 coatings was measured, the coated plates were divided into three sets with thicknesses as close as possible. After that, the epoxy and polyurethane paints were applied to the P3 coatings to prepare the P1 and P2 coatings. 

#### 2.2.1. Material Characterization

The obtained coatings (Figure 1) were characterized for dielectric and mechanical properties before and after their exposure to various climatic factors, including dry heat, UV radiation, and humidity, as well as extreme conditions such as salt fog, alpine atmosphere, and marine atmosphere. Additionally, the remaining lifetime, until the coating before replacement is necessary, was determined due to degradation risks of potential equipment failures. The interpretation of the obtained results involved studying the variations in the tangent of the dielectric loss angle (tg δ) based on the applied frequency.

##### Dielectric Characterization

The real (ε′) and imaginary parts (ε″) of relative permittivity, along with the tangent of dielectric loss angle (tg δ = ε″/ε′) of the P1–P3 coatings, were determined using dielectric spectroscopy with a Solartron 1260A dielectric spectrometer (Solartron Analytical, Farnborough, UK). The measurements were conducted with an AC voltage amplitude of 3 V over a frequency range of 1–10 kHz, using a measuring electrode with a diameter of 30 mm.

##### Dry Heat

The exposure of the P1–P3 coatings to dry heat was conducted using a Memmert universal oven, model UF30, with forced convection. The temperatures and exposure cycles were as follows:

- A temperature of 100 °C for 240 h, 480 h, 720 h, and 960 h, with intermediate tg δ measurements being taken after each cycle;
- A temperature of 200 °C for 72 h, 144 h, 216 h, and 288 h, with intermediate tg δ measurements being taken after each cycle;
- A temperature of 250 °C for 24 h, 48 h, 72 h, and 96 h, with intermediate tg δ measurements being taken after each cycle.

The remaining lifetime [50] until the polymeric coating needs replacement was determined to prevent material damage resulting from the failure or permanent non-functionality of equipment that includes these polymeric coatings. A set of P1, P2, and P3 coated steel plates was placed in a Memmert oven at a temperature of 100 °C for this determination. After 120 h, the plates were removed, cooled to room temperature, and the tg δ was measured. This heating and cooling cycle was repeated 14 times. The temperature resistance of P1–P3 coatings was assessed by analyzing the variation in tg δ and electrical resistivity with frequency to estimate the critical resistivity and time to degradation. The critical value of electrical resistivity, indicating material degradation, was determined when the electrical resistivity decreased to 30% of its initial value [50]. The temperature of 100 °C used in accelerated aging was selected as it is higher than the operating temperature of the equipment incorporating these polymeric protective coatings.

##### Salt Fog

The study of the P1–P3 coatings to the action of salt fog was conducted using an SP 500 type salt fog test cabinet specifically designed for long-term testing duration (≥200 h). The equipment featured a hot bubble tower for humidifying the compressed air before it reached the mist atomizer. Automatic water filling through a designated port was included, along with an external 90 L mineral water tank for the saltwater solution. The additional tank capacity reduced the changeover time during prolonged testing, while an integrated filter, along with the existing one on the supply line of the tank, prevented the accumulation of salt crystals that could block the exhaust nozzle. Furthermore, greater thermal efficiency was ensured by a non-transparent heat-insulating coating. The test conditions included a temperature of 36.5 °C, a NaCl concentration of 5 ± 0.5 wt.%, a pH ranging from 6.5 to 7.2, and exposure durations of 200 h and 600 h.

##### UV Radiation and Humidity

The P1–P3 polymeric coatings were subjected for 48–449 h to UV radiation exposure at 100 W/m^2^ and a relative humidity of 60% using the XENOTEST^®^ 220/440 equipment manufactured by Atlas Material Testing Technology LCC., Linsengericht, Germany. The tests were conducted following ASTM standards G151 [51], G155 [52], and D2565 [53]. 

##### Alpine and Marine Atmosphere

The P1 and P2 coatings were stored in real environmental conditions during the alpine and marine atmosphere tests. 

The samples were exposed for 880 days to the alpine atmosphere, with intermediate tg δ measurements being taken after 86 days and 210 days at an altitude of 1246 m above sea level in the Gutâi Mountains, Maramureș county, Romania [54], specifically at Mogoşa Peak [55]. Cooler temperatures, typically ranging from 10 °C to 26 °C in the summer and from −10 °C to 0 °C in the winter, higher precipitation, humidity, and wind levels, and reduced atmospheric pressure due to the elevation, compared to lower altitudes, were included in the alpine climate conditions. 

The exposure of the P1 and P2 coatings to the marine atmosphere was conducted from the Gloria offshore drilling rig [56,57,58] located in the Black Sea, Romania, for 244 days. Interim tg δ measurements were taken after 70 days and 154 days. The P1 and P2 coatings were fully immersed in the Black Sea water at a shallow depth during the summer-winter. The Black Sea water was observed to exhibit a typical sea surface temperature of 9–10 °C during the winter and 24–25 °C during the summer and a sea surface salinity of about 17–18 ‰ (parts per thousand), which is equal to 17–18 PSU (Practical Salinity Units) during the summer–autumn and 19 ‰ or 19 PSU during the winter [57,59]. The wave data specific to the Gloria drilling rig were reported elsewhere [58].

##### Mechanical Characterization

The indentation hardness (H_IT_), Vickers hardness, indentation elastic modulus (E_IT_), and scratch resistance of the initial P1 and P2 paint coatings, as well as those after 600 h of exposure to salt fog (P1-SF and P2-SF paint coatings), were determined using a Micro-Combi Tester (MCT^2^) equipped with a Nano Head Tester (NHT^2^) and a Micro Scratch Tester (MST^2^) from CSM Instruments, Peseux, Switzerland. The nanoindentation testing of both coatings and the steel substrate was conducted using the NHT^2^ module with a diamond Berkovich indenter, following the measurement conditions and Oliver and Phar method described elsewhere [60,61,62]. A Poisson’s ratio (ν) of 0.35 [63,64] was assumed for the polymeric coatings, and ν = 0.3 [61] for the steel substrate. Five nanoindentations were performed per coating and steel substrate, with mean values and standard deviations (SD) being calculated.

Microscratch testing of the P1, P2, P1-SF, and P2-SF paint coatings was carried out using the MCT^2^ equipment and the MST^2^ module, which included a diamond Rockwell indenter with a 200 μm radius. A scratch length of 5 mm was involved in these tests with a three-pass approach: (i) the coating surface was pre-scanned with a 0.03 N load to measure the initial surface profile (P_f_); (ii) a scan was performed with progressive loading from 0.03 N to 20 N at a loading rate of about 30 N/min and a scratching speed of 7.5 mm/min to measure the penetration depth (P_d_) during the linear scratch (P_sc_), calculated as the difference between P_f_ and P_sc_; and (iii) a post-scan of the scratch track was conducted with a 0.03 N load to measure the residual depth (R_d_), calculated as the difference between P_f_ and R_sc_. The optical critical loads (L_c_) at which visible damages occurred on the coatings were determined through optical microscopy of the coating surface along the scratch tracks, using a 20× objective attached to the MCT^2^ equipment, and optical images were captured with the Panorama data acquisition software. 

Mechanical testing through nanoindentation and microscratch was conducted at a room temperature of 25 ± 2 °C and a relative air humidity of 37 ± 2%.

## 3. Results and Discussion

### 3.1. Initial Characterization of the P1, P2, and P3 Coatings

**Figure 2:**
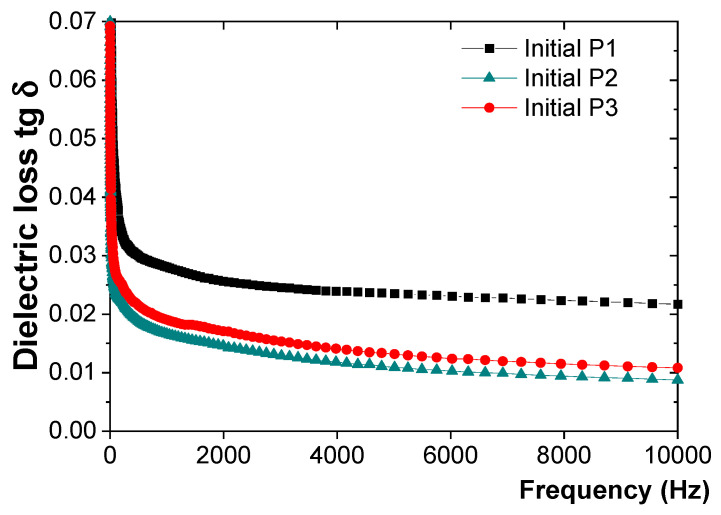
Variation in tg δ with frequency for the initial P1, P2, and P3 coatings.

The variation in tg δ with frequency for the initial coatings is shown in Figure 2. 

It was found that the best insulator is P2, followed by P1 and P3. The P3 plate is covered only with epoxy primer, while the other two are covered with an intermediary epoxy primer layer followed by a top layer of epoxy paint (P1) or polyurethane paint (P2). When comparing the two types of coated plates (P1 and P2), it is evident that the polyurethane paint coating (P2) provides better insulation characteristics than the epoxy paint coating (P1). This is confirmed by the variation in tg δ with frequency, with the P2 paint coating exhibiting an 11.4% lower tg δ than the P1 paint coating.

### 3.2. Dry Heat

Dry heat tests were performed at temperatures of 100 °C, 200 °C, and 250 °C.

#### 3.2.1. Characterization of the Polymeric Coatings to the Action of Dry Heat at 100 °C

**Figure 3:**
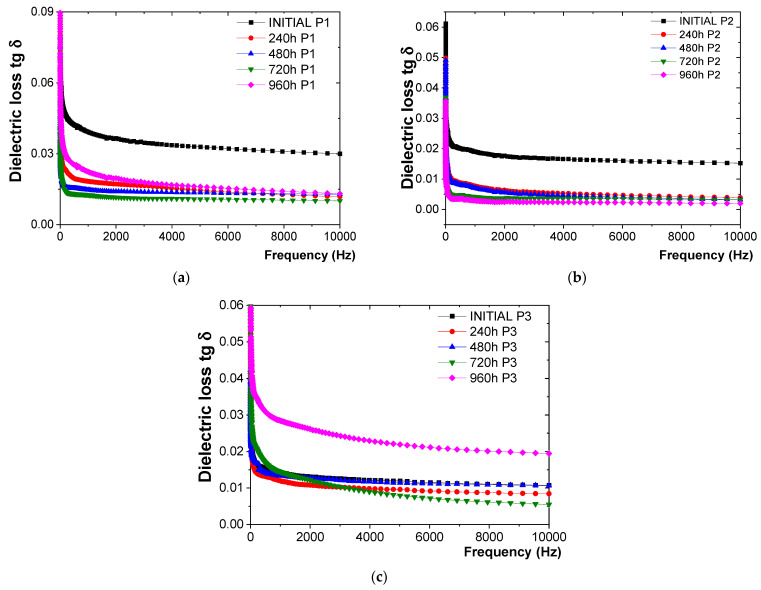
Variation in tg δ with frequency for (**a**) P1, (**b**) P2, and (**c**) P3 coatings before and after up to 960 h of exposure to dry heat at 100 °C.

The response of the P1, P2, and P3 coatings to the influence of dry heat at 100 °C, as observed through the variation in tg δ with frequency, is summarized in Figure 3.

Based on the tg δ measurements (Figure 3), the following observations were made:

- For the P1 coating (Figure 3a), the tg δ values exhibited a relatively constant decreasing trend, averaging about 74% reduction, up to 720 h of exposure to 100 °C. This trend likely reflects the failure of crosslinking reactions, leading to the epoxy resin transitioning into a three-dimensional state. Between 720 h and 960 h of exposure, a significant increase in tg δ values was observed, with values higher by about 63% compared to those recorded at 720 h. However, these values did not exceed the initial value, indicating that the degradation of the polymer coating has not yet occurred.
- For the P2 coating (Figure 3b), the tg δ values tended to decrease relatively constantly up to 960 h of exposure to 100 °C. This trend suggested the completion of polymerization and crosslinking reactions, leading to the polyurethane resin transitioning into a three-dimensional (3D) state. Moreover, no signs of degradation of the polyurethane resin coating (P2) were observed, unlike the epoxy coating (P1).
- For the P3 coating (Figure 3c), since only the protective epoxy primer coating was present, the measured tg δ values exhibited greater dispersion, with fluctuations up to 720 h of exposure to 100 °C. These variations likely originated from pores and voids in the polymer material directly exposed on the metal plates, consistent with comparative measurements taken prior to exposure to dry heat. After 960 h of exposure, an increase of about 45% was observed compared to the value at 720 h and about 100% compared to the initial value of tg δ, indicating the onset of polymer coating degradation.

#### 3.2.2. Characterization of the Polymeric Coatings to the Action of Dry Heat at 200 °C

**Figure 4:**
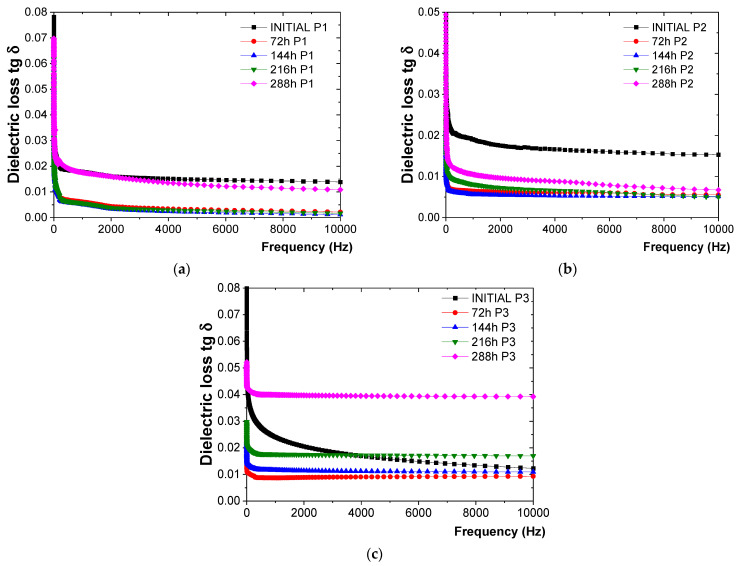
Variation in tg δ with frequency for (**a**) P1, (**b**) P2, and (**c**) P3 coatings before and after up to 288 h of exposure to dry heat at 200 °C.

The response of the P1, P2, and P3 protective coatings to the influence of dry heat at 200 °C, as observed through the variation in tg δ with frequency, is shown in Figure 4.

From the tg δ measurements (Figure 4), the following observations were made:

- For the P1 coating (Figure 4a), the tg δ values tended to decrease relatively constantly up to 216 h of exposure to 200 °C. This trend was likely due to the completion of crosslinking reactions, resulting in the epoxy resin transitioning into a three-dimensional (3D) state. After 216 h of exposure, a constant increase in tg δ values was observed, with an increase of 40% compared to the values obtained after 216 h of exposure, although it did not exceed the initial value of tg δ.
- For the P2 coating (Figure 4b), the tg δ values decreased up to 144 h of exposure to 200 °C, also likely due to the completion of crosslinking reactions leading to a three-dimensional (3D) state of the polyurethane resin. Subsequently, an increase of approximately 62% was observed at 288 h of exposure compared to the previous cycle (216 h), without exceeding the initial value of tg δ.
- For the P3 coating (Figure 4c), the tg δ values decreased up to 72 h, after which they started to increase slightly. After 288 h of continuous exposure, an increase of 111% was observed compared to the previous cycle (216 h), along with an increase of about 10% compared to the initial value of tg δ, indicating the beginning of coating degradation.

#### 3.2.3. Characterization of the Polymeric Coatings to the Action of Dry Heat at 250 °C

**Figure 5:**
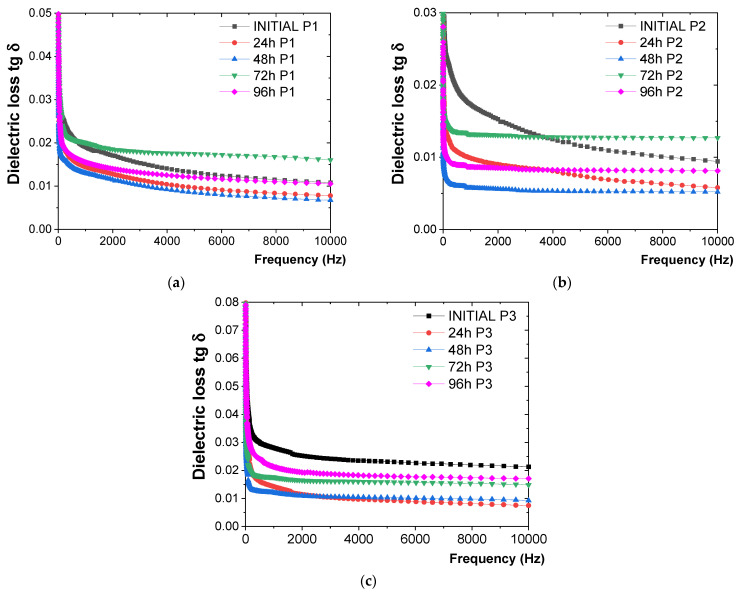
Variation in tg δ with frequency for (**a**) P1, (**b**) P2, and (**c**) P3 coatings before and after up to 96 h of exposure to dry heat at 250 °C.

The behavior of the P1, P2, and P3 coatings under the influence of dry heat at 250 °C, as observed through the variation in tg δ with frequency, is illustrated in Figure 5.

From the tg δ measurements (Figure 5), the following observations were made:

- For the P1 coating (Figure 5a), a continuous decrease in tg δ values was exhibited up to 48 h of exposure to 250 °C. This trend was likely due to the completion of crosslinking reactions, resulting in the epoxy resin transitioning into a three-dimensional (3D) state. However, from 48 h to 72 h, coating degradation began, as evidenced by a sudden increase in tg δ values of about 35% at 72 h compared to those recorded after 48 h of exposure.
- For the P2 coating (Figure 5b), a decrease in tg δ values was observed up to 48 h of exposure to 250 °C. After 48 h, the tg δ values began to increase continuously, indicating that the polymeric coating degradation started after 96 h of exposure.
- For the P3 coating (Figure 5c), greater dispersion in tg δ values was measured, with fluctuations up to 48 h of exposure to 250 °C. These variations were likely due to pores and voids in the polymeric material directly applied to the metal plates, consistent with comparative measurements made before exposure to dry heat. After 48 h of exposure, a constant increase in tg δ values was observed, indicating that the polymeric coating degraded after 96 h of exposure.

#### 3.2.4. Determining the Remaining Lifetime Until the Replacement of the Polymeric Protective Coatings

To provide comprehensive information to users of these polymeric coatings, the remaining lifetime of the coating, until recoating is necessary, must be determined without causing temporary or permanent defects to the equipment it protects. Effective maintenance schedules can be planned by users based on this information, ensuring continuous protection of their equipment and preventing potential damage due to degraded coatings.

**Figure 6:**
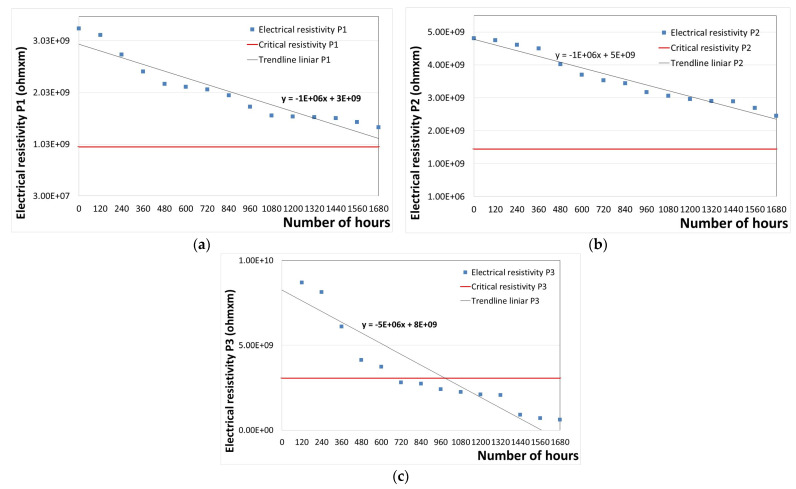
Variation in the electrical resistivity of coatings versus the exposure time at 100 °C: (**a**) P1; (**b**) P2; (**c**) P3.

The variation in the electrical resistivity of P1–P3 coatings versus the exposure time at a temperature of 100 °C is illustrated in Figure 6.

First-degree equations were used to calculate the solutions (x values) of the equations depicted in each graph shown in Figure 6, and the data presented in Table 1 were obtained. This allows for the easy identification of the number of hours of thermal aging [50].

According to the results presented in Table 1, the replacement time of polymeric coatings when irreparable damage occurs can be estimated. It was found that the replacement time is up to 2020 operating hours for the P1 coating, up to 3558 h for the P2 coating, and up to 958 h for the P3 coating. These values are based on the thermal exposure of the coatings according to the standard SR EN 60216-3 [65].

From these data, it can be concluded that the P3 coating degrades the fastest, which can be attributed to the fact that it is covered only with an epoxy primer layer. In contrast, the epoxy (P1) and polyurethane (P2) paint coatings exhibit increased lifetimes. When comparing the two types of top paint coatings, it was found that polyurethane paint (P2) provides a longer lifetime than epoxy paint (P1).

### 3.3. Salt Fog

**Figure 7:**
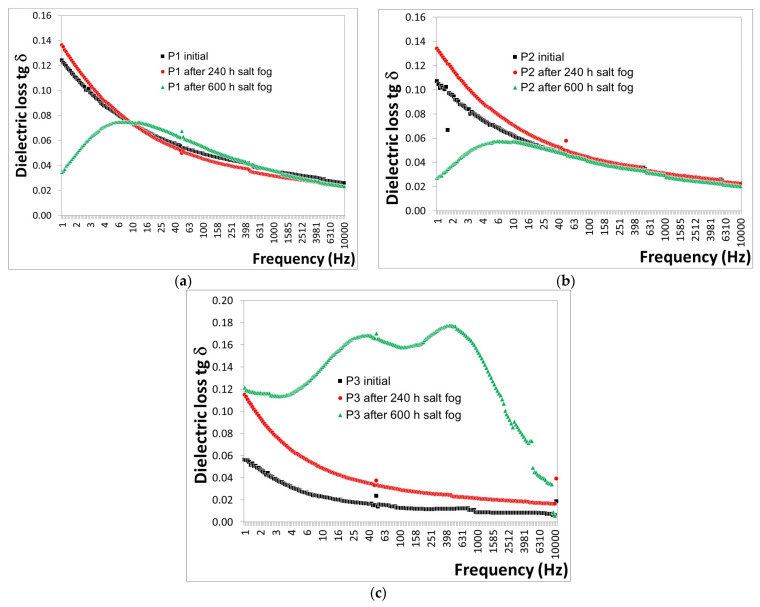
Variation in tg δ with frequency for (**a**) P1, (**b**) P2, and (**c**) P3 coatings before and after 240 h and 600 h of exposure to salt fog.

The behavior of salt fog exposure for 240 h and 600 h, as expressed by the variations in tg δ with frequency for P1–P3 coatings, is illustrated in Figure 7.

Based on the tg δ measurements (Figure 7), the following observations were made:

- For the P1 coating (Figure 7a), a decrease in tg δ values of approximately 5.24% was observed after 240 h, followed by a slight increase of approximately 0.7% after 600 h of exposure to salt fog.
- For the P2 coating (Figure 7b), a decrease in tg δ values of approximately 15.4% was observed after 240 h, with a further decrease of approximately 23.5% after 600 h of exposure to salt fog.
- For the P3 coating (Figure 7c), a decrease in tg δ values of approximately 109.8% was observed after 240 h, and a further decrease of approximately 615.7% after 600 h of exposure to salt fog.

Among the three polymeric coatings, it was found that degradation occurred in P3 after 600 h of exposure to salt fog, as evidenced by the sharp increase in tg δ. When P1 and P2 were compared, degradation was observed to have begun in P1, indicated by the slight increase in tg δ after 600 h of exposure to salt fog, which was not observed in P2, suggesting that degradation in P2 started later. This led to the conclusion that the P2 coating exhibits greater resistance to salt spray action compared to the P1 coating.

**Figure 8:**
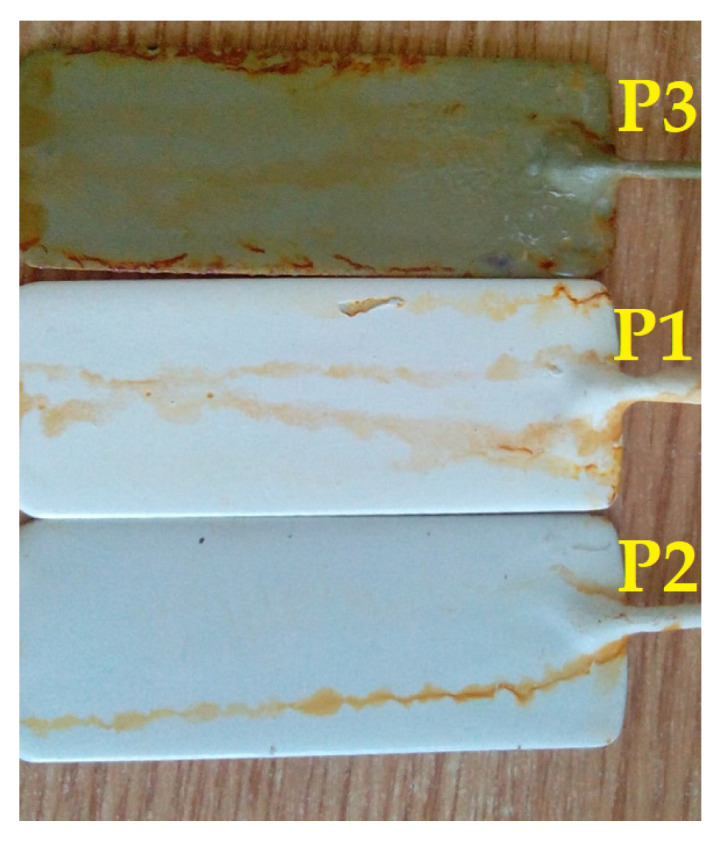
Visual examination of P1, P2, and P3 coatings after 600 h of exposure to salt fog.

After 600 h of exposure to salt fog, visible surface cracks were observed in all three types of polymeric coatings (as confirmed by visual examination highlighted in Figure 8), with the P2 coating being the least affected.

The tg δ values measured on the surface of P1, P2, and P3 coatings after 600 h of exposure to salt fog are no longer accurately reflective of the intrinsic properties of the polymeric coatings. This is due to the influence of the exposed metal surface of the plates, which results from the cracking of the polymeric coatings, leading to random variations in the tg δ measurements.

### 3.4. Characterization of the Behavior of Polymeric Protective Coatings to UV Radiation and Humidity

**Figure 9:**
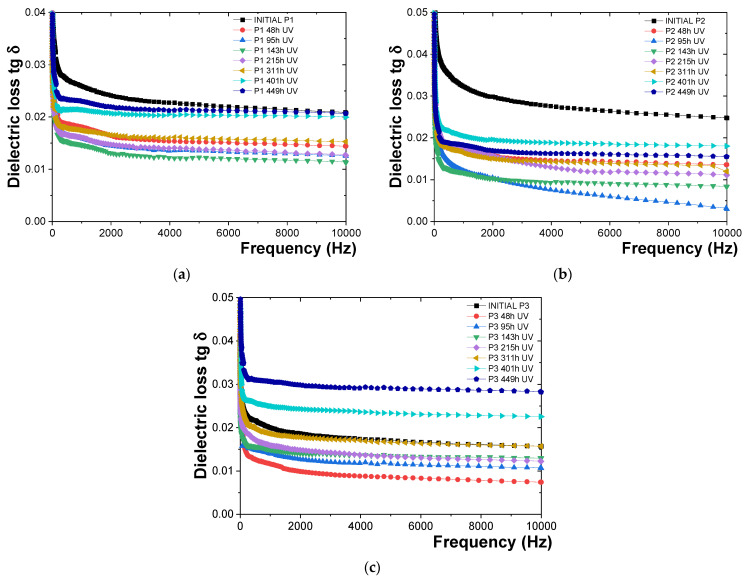
Variation in tg δ with frequency for (**a**) P1, (**b**) P2, and (**c**) P3 coatings before and after up to 449 h of exposure to UV radiation.

The behavior of P1, P2, and P3 polymeric protective coatings in terms of variation in tg δ with frequency is illustrated in Figure 9. The samples were exposed up to 449 h to UV radiation of 100 W/m^2^ and a relative humidity of 60%.

Based on the tg δ measurements (Figure 9), the following observations were made:

- For the P1 polymeric coating (Figure 9a), the tg δ values measured after exposure to UV radiation decreased compared to the initial tg δ values by about 28.43% for the 48 h dose, 30.48% for the 95 h dose, 36.69% for the 143 h dose, 37.86% for the 215 h dose, 29.09% for the 311 h dose, 26.48% for the 401 h dose, and 15.86% for the 449 h dose. It was observed that the protective epoxy coating (P1) did not degrade after 449 h of exposure to UV radiation and humidity. The onset of degradation of the epoxy resin coating (P1) was identified by a sharp and continuous increase in tg δ values.
- For the P2 polymeric coating (Figure 9b), it was found that the tg δ values decreased compared to the initial tg δ values by about 41.29% for the 48 h dose, 45.28% for the 95 h dose, 52.7% for the 143 h dose, 35.50% for the 215 h dose, 40.64% for the 311 h dose, 39.80% for the 401 h dose, and 39.75% for the 449 h dose. The same behavior in terms of tg δ values as observed for the epoxy resin-based polymeric coating (P1) was indicated by these results. It was concluded that the onset of degradation for the polyurethane resin was not reached after 449 h of exposure to UV radiation and humidity, as evidenced by the absence of a sharp and continuous increase in tg δ values.
- For the P3 polymeric coating (Figure 9c), it was observed that the tg δ values initially decreased compared to the initial tg δ values upon exposure to UV radiation, then increased. Specifically, tg δ values were reduced by approximately 6.44% for the 48 h dose, 5.71% for the 95 h dose, 30.35% for the 143 h dose, and 23.95% for the 215 h dose. Subsequently, a slight increase was recorded, with tg δ values rising by about 2.56% for the 311 h dose, 3.17% for the 401 h dose, and an increase of about 48.69% for the 449 h dose, indicating that the epoxy primer coating has begun to deteriorate.

**Figure 10:**
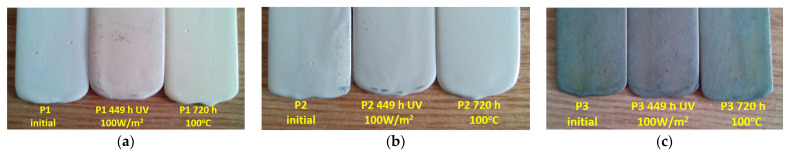
Appearance of (**a**) P1, (**b**) P2, and (**c**) P3 polymeric coatings before and after 449 h of exposure to UV radiation of 100 W/m^2^ and relative humidity of 60%, and 720 h of exposure to dry heat at 100 °C.

The aspect of the polymeric coatings before and after exposure to UV radiation and humidity for 449 h and to dry heat at 100 °C for 720 h is shown in Figure 10.

The results obtained (Figure 9) indicate that similar behavior in terms of tg δ values was observed for all analyzed polymeric coatings. After 449 h of exposure to UV radiation and humidity, no onset of degradation for the epoxy resin was observed, which would be indicated by a sharp and continuous increase in tg δ values. However, it was found that the polyurethane coating (P2) degrades more slowly compared to the epoxy coating (P1) over the same exposure durations. Therefore, the polyurethane coating (P2) is considered more resistant to UV radiation and moisture than the epoxy coating (P1).

### 3.5. Characterization of the Behavior of the Protective Coatings in Extreme Conditions—Alpine and Marine Atmosphere

The tests in the alpine and marine atmospheres were conducted exclusively on the epoxy and polyurethane polymeric coatings (P1 and P2) applied over epoxy primer coating/steel plates and not on the epoxy primer coating (P3). This approach was chosen to specifically study the behavior of the top layers of polymeric protective coatings under extreme conditions.

#### 3.5.1. Alpine Atmosphere

**Figure 11:**
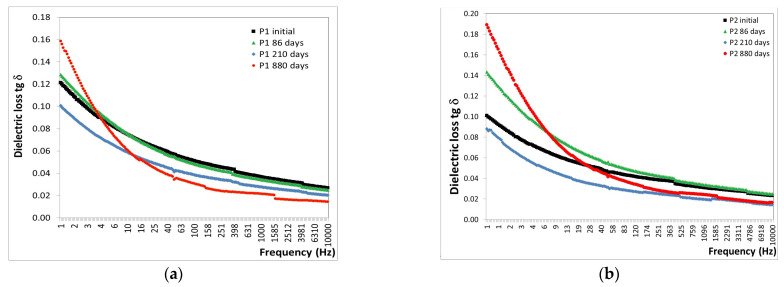
Variation in tg δ with frequency for (**a**) P1 and (**b**) P2 coatings before and after up to 880 days of exposure to alpine atmosphere.

The behavior of the polymeric epoxy and polyurethane coatings (P1 and P2) subjected to the alpine atmosphere for 0, 86, 210, and 880 days, from the point of view of the variation in tg δ with frequency, is shown in Figure 11. 

Based on the results shown in Figure 11, the findings are as follows:

- After 86 days of exposure to the alpine atmosphere, a slight decrease in tg δ values was observed, with a reduction of approximately 1% for both P1 and P2 coatings.
- After 210 days of exposure to the alpine atmosphere, a continued decrease in tg δ values was noted, with reductions of approximately 25% for the P1 coating and 39% for the P2 coating.
- After 880 days of exposure to the alpine atmosphere, a decrease in tg δ values of 19% was recorded for the P1 coating and 13% for the P2 coating.

#### 3.5.2. Marine Atmosphere

**Figure 12:**
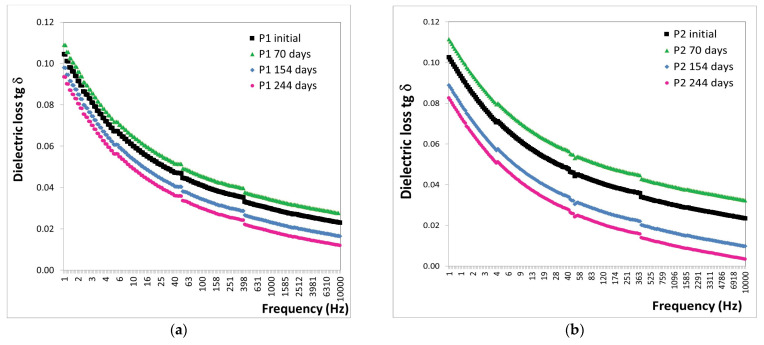
Variation in tg δ with frequency for (**a**) P1 and (**b**) P2 coatings before and after up to 244 days of exposure to marine atmosphere.

The behavior of the P1 and P2 polymeric coatings subjected to the marine atmosphere for 0, 70, 154, and 244 days was analyzed based on the variation in tg δ with frequency. This analysis is presented in Figure 12. 

The analysis of the tg δ results (Figure 12) revealed the following observations:

- After 70 days of exposure to the marine atmosphere, significant increases were observed in the tg δ values, with an 88% increase for the P1 coating and a 91% increase for the P2 coating.
- After 154 days of exposure to the marine atmosphere, average decreases in tg δ values were noted, approximately 14% for the P1 coating and 27% for the P2 coating.
- After 244 days of exposure to the marine atmosphere, average decreases in tg δ values were observed, approximately 45% for the P1 coating and 54% for the P2 coating.

The trend of decreasing tg δ values with increasing exposure duration to the marine atmosphere in the Black Sea water indicates that the degradation limit of the polymeric coatings has not yet been reached.

These analyses, conducted under extreme conditions over extended periods, did not reach the point of degradation within the available timeframe. It can be concluded that the polymeric coatings endured very harsh conditions, withstanding 880 days in the alpine atmosphere and 244 days in the marine atmosphere. 

**Figure 13:**
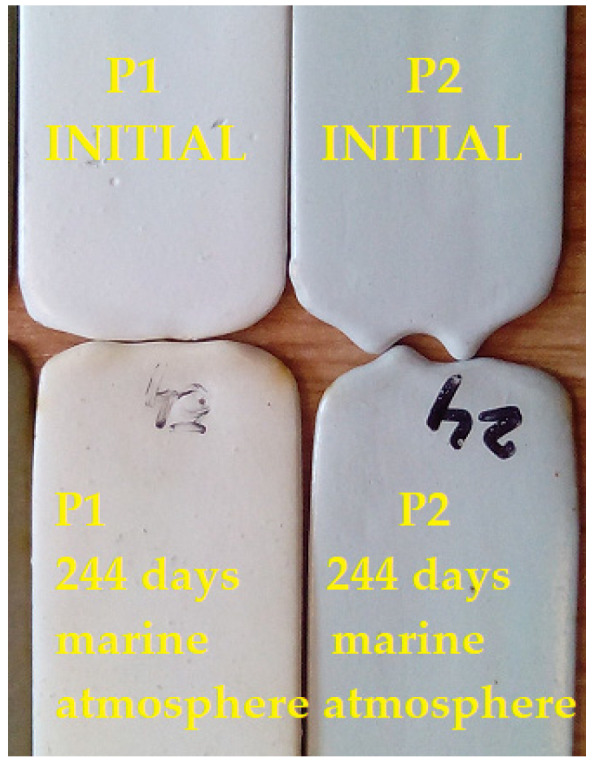
Aspect of P1 and P2 anticorrosive protective coatings before and after 244 days of exposure to marine atmosphere.

The aspect of the P1 and P2 coatings after 244 days of exposure to the marine atmosphere is shown in Figure 13.

### 3.6. Mechanical Characterization

Mechanical characteristics of the paint coatings and steel substrate, determined using nanoindentation testing and the Oliver and Phar method [62], are summarized in Table 2.

The nanoindentation data in Table 2 revealed that all polymeric paint coatings exhibited lower mechanical characteristics compared to the steel substrate. The indentation hardness (H_IT_) and Vickers hardness of the coatings were found to increase in the order: P2-SF coating, P2 coating, P1-SF coating, and P1 coating. Consequently, the P1 coating demonstrated the best resistance to irreversible (plastic) and reversible (elastic) deformation [66], followed by the P1-SF coating, P2 coating, and P2-SF coating. Furthermore, the highest H_IT_ values for the P1 coating indicate superior wear resistance compared to the other coatings. However, the variation in H_IT_ and Vickers hardness values for both types of polymeric coatings was observed to be low.

The indentation modulus (E_IT_), which approximates the elastic modulus [67], was found to be 30.7–35.7 times higher for the steel substrate than for the coatings. Higher E_IT_ values were observed for the P2 and P1 coatings, indicating greater resistance to elastic deformation [66] compared to the P2-SF and P1-SF coatings, which had been exposed to salt fog. However, similar to hardness, the variation in E_IT_ values for both types of polymeric coatings was found to be low.

The H_IT_/E_IT_ ratio, also known as true hardness [66], was found to be higher for all coatings (0.0337–0.0434) compared to the steel substrate (0.0176). The true hardness of the coatings increased in the order: P2-SF coating, P2 coating, P1-SF coating, and P1 coating. A higher true hardness in the coatings suggests better resistance to plastic deformation and improved wear resistance [66]. Both the P1 and P1-SF epoxy coatings exhibited higher true hardness than the P2 and P2-SF polyurethane coatings. However, the small H_IT_/E_IT_ ratio, considered an “elasticity index” [66], indicates a predominance of permanent deformation in both the coatings and the steel substrate. Moreover, greater resistance to plastic deformation was proved by the P1 and P1-SF coatings compared to the P2 and P2-SF coatings. This finding was confirmed by the higher H_IT_^3^/E_IT_^2^ ratio (0.00029–0.00032 GPa) of the P1 and P1-SF coatings, compared to the H_IT_^3^/E_IT_^2^ ratio of 0.00014–0.00019 GPa for the P2 and P2-SF coatings. Additionally, the steel substrate exhibited the best resistance to plastic deformation, as suggested by the highest H_IT_^3^/E_IT_^2^ ratio of 0.00070 GPa.

The results presented above indicate that the epoxy paint coatings exhibit slightly greater mechanical characteristics compared to the polyurethane paint coatings. This behavior was maintained even after 600 h of salt fog testing. Additionally, the mechanical properties of the epoxy and polyurethane paint coatings applied on steel substrate are comparable to the hardness and elastic modulus values of similar coatings reported in the literature [63,64,68,69,70].

**Figure 14:**
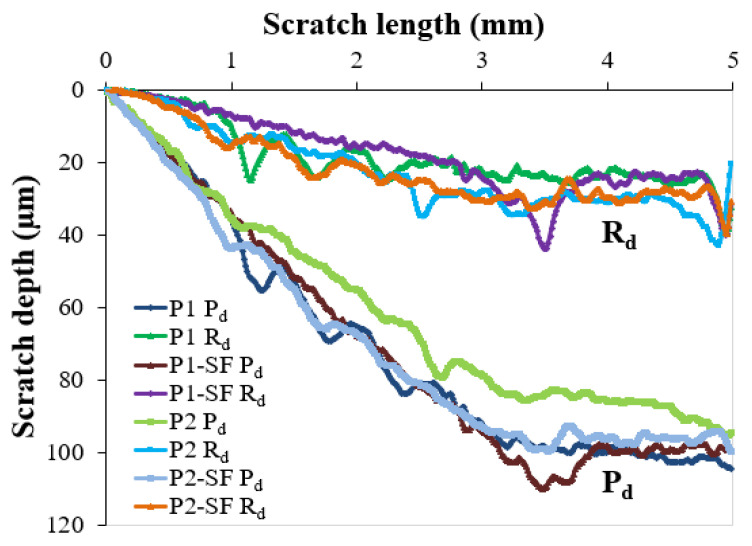
P_d_ and R_d_ plots for the tested paint coatings over a scratch length of 5 mm.

The plastic and elastic behavior of the polymeric paint coatings was also confirmed by the microscratch results. The plots of penetration depth (P_d_) and residual depth (R_d_) for the tested paint coatings over a scratch length of 5 mm are illustrated in Figure 14.

All coatings exhibited a continuous increase in penetration depth (P_d_) during scratch testing with a linear progressive load from 0.04 N to 20 N (Figure 14). Several fluctuations observed in the P_d_ plots were attributed to local plastic deformation, suggesting damage in the coatings. The slow rise in penetration depth was due to the lower hardness of the paint coatings compared to the steel substrate. The lowest residual depth (R_d_) values, which indicate permanent plastic deformation, were observed in the stable part of the R_d_ plots for the P1 coating, followed by the P1-SF, P2, and P2-SF coatings. Typically, the least amount of permanent plastic deformation and the highest degree of relaxation along a scratch length of 5 mm were exhibited by the P1 epoxy paint coating. This behavior indicates superior scratch resistance of the P1 coating compared to the P2 coating. Conversely, a lower penetration depth compared to the P1 coating was demonstrated by the P2 polyurethane paint coating. This suggests that while the P2 coating is more resistant to initial penetration, it does not perform as well in overall scratch resistance as the P1 coating. However, the differences in P_d_ and R_d_ values between the two types of polymeric coatings remained within a narrow range, even after 600 h of salt fog exposure.

The elastic recovery (ER) was computed using Equation (1) [71]:

ER = (P_d_ − R_d_)/P_d_ (1)

**Figure 15:**
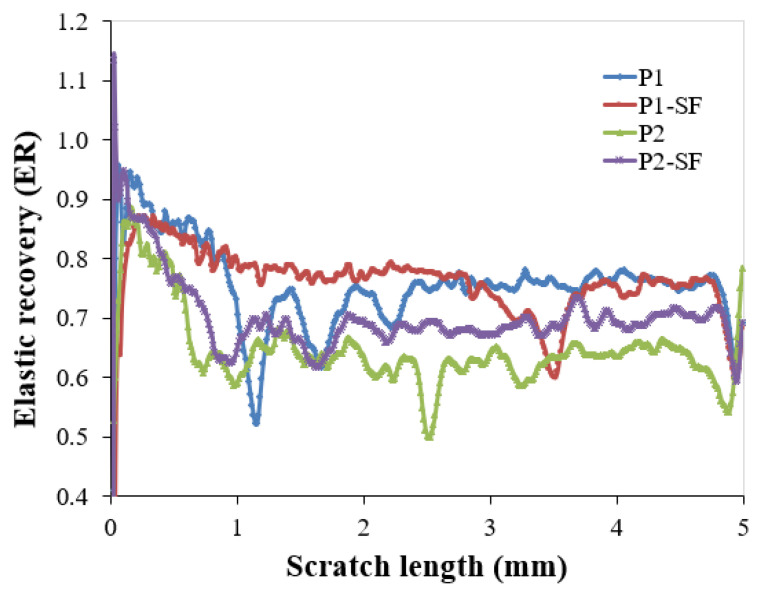
ER plots for the tested paint coatings over a scratch length of 5 mm.

The ER plots for the tested paint coatings over a scratch length of 5 mm are shown in Figure 15. 

The ER values of the coating were found to increase in the order of P1-SF, P2, P2-SF, and P1 in the first part of the scratch length (<0.2 mm). Following this, the P1-SF and P1 coatings exhibited the highest ER values. Moreover, none of the coatings peeled off, indicating good adhesion to the epoxy primer and steel substrate. A similar plastic deformation mechanism was observed for all paint coatings. Initial cracking of the coatings, as determined by optical microscopy, defined the first critical load (L_C1_) at 4.34 N for the P1 coating, 3.84 N for the P1-SF coating, 3.49 N for the P2 coating, and 3.31 N for the P2-SF coating. Subsequently, ductile perforation of the coatings occurred over the rest of the scratch length without exposing the steel substrate. The best microscratch resistance was exhibited by the P1 coating, as suggested by the highest L_C1_ value. However, there was a low variation in L_C1_ values for both types of polymeric coatings. 

The plastic resistance (PR), which indicates the resistance of the scratched coatings to permanent plastic deformation, was computed using Equation (2) [71], where F_n_ < L_C1_:

PR = F_n_/R_d_ (2)

For the calculation of the PR values, a normal load (F_n_) of 2.03 N at a scratch length of 0.5 mm was considered, which is in the stable part of the P_d_ and R_d_ plots shown in Figure 14. The PR values were computed as 0.85 N/μm for the P1 coating, 0.81 N/μm for the P1-SF coating, 0.55 N/μm for the P2 coating, and 0.42 N/μm for the P2-SF coating. The higher PR and lower R_d_ values of the P1 epoxy paint coating compared to the other paint coatings suggest that the P1 coating has superior plastic resistance.

## 4. Conclusions

The findings on the preparation and performance investigation of two anticorrosive protective coatings based on polymeric epoxy and polyurethane resin paints (P1 and P2) applied on an epoxy primer coating/steel plates (P3) when subjected to various accelerated degradation factors are presented in this study. The effectiveness of commercial epoxy and polyurethane paints, combined with a multi-layered approach, was demonstrated for industrial steel substrates by enhancing adhesion and corrosion resistance.

The experimental results are summarized as follows:

- Dry heat: The coatings underwent stages of complete crosslinking and degradation. At 100 °C, only the P3 coating degraded after 1968 h. At 200 °C, the P1 and P2 coatings began degrading after 648 h, with degradation completing at 250 °C. The P2 coating demonstrated the highest temperature resistance among the coatings tested.
- Remaining lifetime: The analysis of the variation in tg δ and electrical resistivity with frequency revealed that a longer service life was provided by the polyurethane coating (P2) compared to the epoxy coating (P1) after exposure to 100 °C for 1680 h.
- Salt spray (fog) resistance: Slight degradation onset was shown by the P1 coating after 600 h of exposure to salt fog (36.5 °C, 5 ± 0.5 wt.% NaCl, and pH = 6.5–7.2), indicated by a minor increase in tg δ values, whereas this early degradation was not exhibited by the P2 coating. Significant degradation was shown by the P3 coating after 600 h. Thus, more resistance to salt fog was exhibited by the P2 coating compared to the P1 and P3 coatings.
- UV and moisture resistance: After 449 h of exposure to UV radiation of 100 W/m^2^ and a relative humidity of 60%, degradation was not reached by any of the polymeric top coatings (P1 and P2), as indicated by tg δ values. Furthermore, slower degradation was observed in the P2 coating compared to the P1 coating, indicating higher resistance to UV radiation and moisture. However, deterioration of the P3 coating began after 449 h of exposure.
- Extreme conditions: Alpine atmosphere tests conducted for 880 days at an altitude of 1246 m and marine atmosphere tests conducted for 244 days in the Black Sea water revealed no degradation for both P1 and P2 coatings under these conditions, as indicated by the decreasing tendency in tg δ values.
- Mechanical properties: Nanoindentation and microscratch tests revealed that lower mechanical characteristics were exhibited by all coatings compared to the steel substrate. Slightly greater hardness (H_IT_ of 0.168 ± 0.011 GPa), a lower elastic modulus (E_IT_ of 3.867 ± 0.168 GPa), and superior wear and scratch resistance were shown by the P1 coating compared to the P2 coating (H_IT_ of 0.149 ± 0.006 GPa, and E_IT_ of 4.185 ± 0.100 GPa), under the tested conditions. These differences remained within a narrow range even after 600 h of exposure to salt fog.

Superior protection was provided by both the P1 and P2 top coatings compared to the intermediary epoxy primer coating (P3) alone, highlighting the effectiveness of the additional top layers. The study demonstrated that significant durability and resistance were exhibited by both the epoxy-based (P1) and polyurethane-based (P2) top coatings when subjected to accelerated degradation tests, including dry heat and salt spray.

Two polymeric paint coatings (P1 and P2) were characterized for their potential use as anticorrosive coatings under various conditions in this work. Based on the experimental data obtained, it can be concluded that strengths were exhibited by both coatings depending on the conditions. The polyurethane resin coating (P2) is recommended for environments requiring higher resistance to temperature, UV radiation, moisture, and salt fog. Conversely, the epoxy resin coating (P1) demonstrated slightly better mechanical properties and may be suitable for applications where these characteristics are essential.

## Figures and Tables

**Table 1:** Results for determining the critical electrical resistivity and time at which P1–P3 coatings can be considered as degraded after thermal aging at 100 °C.

Sample	Equation	y	b	a	Lifetime until Complete Degradation (h)
P1	y = −1 × 10^6^x + 3 × 10^9^	9.80 × 10^8^	3.00 × 10^9^	−1.0 × 10^6^	2020
P2	y = −1 × 10^6^x + 5 × 10^9^	1.44 × 10^9^	5.00 × 10^9^	−1.0 × 10^6^	3558
P3	y = −5 × 10^6^x + 8 × 10^9^	3.06 × 10^9^	8.00 × 10^9^	−5.0 × 10^6^	988

**Table 2:** Mechanical characteristics of the paint coatings and steel substrate were determined using nanoindentation testing and the Oliver and Phar method.

Sample	Mean H_IT_ ± SD (GPa)	Mean HV ± SD	Mean E_IT_ ± SD (GPa)	H_IT_/E_IT_	H_IT_^3^/E_IT_^2^ (GPa)
P1 coating	0.168 ± 0.011	15.5 ± 1.0	3.867 ± 0.168	0.0434	0.00032
P1-SF coating	0.156 ± 0.012	14.4 ± 1.1	3.624 ± 0.171	0.0430	0.00029
P2 coating	0.149 ± 0.006	13.8 ± 0.6	4.185 ± 0.100	0.0356	0.00019
P2-SF coating	0.121 ± 0.009	11.2 ± 0.8	3.591 ± 0.111	0.0337	0.00014
Steel substrate	2.261 ± 0.088	209.4 ± 8.2	128.328 ± 2.518	0.0176	0.00070

## Data Availability

Data are contained within the article.
